# Higher Intake of Total Dietary Essential Amino Acids Is Associated with a Lower Prevalence of Metabolic Syndrome among Korean Adults

**DOI:** 10.3390/nu14224771

**Published:** 2022-11-11

**Authors:** Jihyun Im, Hyoungsu Park, Kyong Park

**Affiliations:** 1 Department of Food and Nutrition, Yeungnam University, Gyeongsan 38541, Korea; 2 R&D Unit, Maeil Health Nutrition Co., Ltd., Pyeongtaek 17714, Korea

**Keywords:** essential amino acid, metabolic syndrome, dietary intake, nutrition requirement, Korea

## Abstract

We hypothesized that a well-balanced intake of total essential amino acids (EAAs) may be associated with lower prevalence of metabolic syndrome among Korean adults. This population-based cross-sectional study included 25,787 participants aged ≥30 years from the 2008–2019 Korea National Health and Nutrition Examination Survey. Dietary information was obtained from 24 h recall data. Demographic and lifestyle factors were assessed using self-administered questionnaires, and metabolic biomarkers were obtained from a health examination. Total essential amino acid score (EAAS) was calculated to determine whether essential amino acid (EAA) intake meets the recommended nutrient intake (RNI). Multivariable-adjusted odds ratios (ORs) and 95% confidence intervals (CIs) were estimated using logistic regression models. After adjusting for multiple confounding factors, participants with higher EAAS had a significantly lower prevalence of high blood pressure (OR: 0.86, 95% CI: 0.75–0.98), hypertriglyceridemia (OR: 0.86, 95% CI: 0.76–0.98), and Metabolic syndrome (MetS) (OR: 0.86, 95% CI: 0.74–0.996). Spline regression analysis confirmed linearity of the association between total EAAS and MetS. EAA intake and MetS are associated with an inverse dose–response relationship in which metabolic disease may be prevented when the overall EAA intake meets the RNI.

## 1. Introduction

Metabolic syndrome (MetS) is often defined as a combination of several risk factors related to cardiovascular diseases [1]. According to the data published by the Korean Society of Cardiometabolic Syndrome in “Metabolic Syndrome Fact Sheet in Korea 2021”, the prevalence of MetS in Korea has been steadily increasing over the past 12 years (21.6% in 2007; 22.9% in 2018) [2]. In a report analyzing data from the 2016–2018 Korea National Health and Nutrition Examination Survey (KNHANES), the prevalence of MetS was 7.1% in people aged 20–29 years, and 17.1% in those aged 30–39 years, showing a sharp increase in people in their thirties, and a trend that continued to increase with age [2]. If MetS is left untreated for a long time, it can increase the risks of coronary heart disease and stroke, as well as result in increasing socio-economic burden due to increased medical expenses [3,4].

Recent studies have investigated the association between amino acid intake and metabolic disorders [5,6,7,8,9,10]. However, most of these studies were short-term clinical trials with a small sample size [5,6], or were focused on the effect of individual amino acids with inconsistent results [7,8,9,10], only suggesting simplified intake levels for comparison. In fact, the intake range of amino acids in the population varies greatly between countries and ethnicities. For example, Koreans traditionally consume high amounts of carbohydrates due to their rice-based diet, therefore, their intake of protein, especially animal protein, is relatively low compared with Western countries [11]. Therefore, rather than calculating the relative risk according to the absolute intake range of amino acids in each study population, it is important to evaluate the extent to which essential amino acid (EAA) intake levels meet the recommended nutrient intake (RNI), and whether satisfactory levels are associated with metabolic health conditions.

We hypothesized that a well-balanced intake of EAA may be associated with lower prevalence of MetS among Koreans whose animal protein intake is relatively low. To test this hypothesis, we evaluated Korean adults’ EAA intake levels by calculating a score that computes the achieved intake levels relative to the Dietary Reference Intakes for Koreans (KDRIs) and examined the association between the EAA score (EAAS) and MetS using data from the 2008–2019 KNHANES, a large-scale cross-sectional study of Koreans.

## 2. Materials and Methods

### 2.1. Study Population

The KNHANES is a large-scale cross-sectional study based on the National Health Promotion Act, which is a statutory survey on people’s health behaviors, chronic disease prevalence, and food and nutrition intake [12]. The KNHANES was conducted at 3-year intervals from 1998 (first survey) to 2005 (third survey); however, from the fourth survey onwards (2007–2009), the system was revised to include annual surveys to improve the timely reporting of national statistics. At present, eightth survey have been conducted, the latest being completed in 2019 [12]. The KNHANES includes health interviews, nutritional surveys, and health examinations. A detailed description of the data collection and processing methods is provided in a previous study [12].

In this analysis, out of a total of 68,879 participants aged ≥30 years who participated in the 2008–2019 KNHANES, the following participants were excluded from the analysis: those with missing data on survey sampling weights (*n* = 10,706); those with daily total energy intake <500 kcal or >5000 kcal (*n* = 934); those who responded that their reported dietary intake in the 24 h recall survey was different from their usual intake (*n* = 13,733); pregnant or lactating women (*n* = 422); those with missing data on metabolic biomarkers (*n* = 2892); and those with prevalent cancer or severe cerebrovascular and cardiovascular disease at the time of the survey (*n* = 14,405). Our final analysis included 25,787 participants.

The data were collected after obtaining written informed consent from all participants. The survey was approved by the Institutional Review Board (IRB) at the Korea Disease Control and Prevention Agency (approval number: 2008-04EXP-01-C, 2009-01CON-03-2C, 2010-02CON-21-C, 2011-02CON-06-C, 2012-01EXP-01-2C, 2013-07CON-03-4C, 2013-12EXP-03-5C, 2018-01-03-P-A, 2018-01-03-C-A).

### 2.2. Demographic and Lifestyle Information

Demographic and lifestyle data including age, sex, household income, and education level were collected through interviews by trained investigators, while health-related data on alcohol consumption, smoking status, and physical activity level were collected through self-reported questionnaires [12]. The monthly average equivalent household income was calculated considering age and sex, and classified into four categories, “lower”, “mid-low”, “mid-high”, and “higher”. These categories were then reclassified into “lower or mid-low” and “mid-high or higher” categories for the analysis. Education levels were classified as “lower than high school education” and “high school education or higher”. Smoking status was classified as “smokers” if they confirmed daily or occasional smoking, and “non-smokers” if they had been a smoker in the past but not currently smoking. Alcohol consumption was calculated based on the frequency of drinks per week, multiplied by the amount of alcohol consumed at once, with participants classified as “drinkers” if there was an intake, and “non-drinkers” if there was no intake. For physical activity, metabolic equivalents (METs) were calculated by multiplying the duration and frequency of each physical activity per week and assigning values according to the exercise intensity corresponding to each activity, which was calculated as the weekly physical activity level (METs-hour/week) [13]. Physical activity levels were classified into tertiles (low, mid, and high).

### 2.3. Dietary Amino Acid and Total EAAS Calculation

Nutrient intake was calculated based on the information obtained using the 24 h recall method through direct interviews with a trained examiner [12]. Interviews were conducted in the form of a face-to-face survey, in which trained examiners directly interviewed the participants, and supplementary materials were also used to collect specific data on the survey items and increase recall [12]. In this study, the intake of essential amino acids (EAAs) was calculated using 24 h recall data from the 2008–2019 KNHANES.

We used an established amino acid database to calculate the content of EAAs in food [14]. An amino acid database was constructed using the National Standard Food Composition Table 9.2 ver. from the Rural Development Administration [15] and the Computer Aided Nutritional Analysis Program 5.0 from the Korean Nutrition Society [16], and the intake of EAAs was then calculated from protein intake. Information related to the construction of the amino acid database was described in detail by Chae, et al. [14].

A total of nine amino acids were included: leucine, isoleucine, valine, lysine, histidine, threonine, methionine, phenylalanine, and tryptophan. The requirements for intake of EAAs were divided based on the RNI of each EAA recommended by sex and age group in the “2020 Dietary Reference Intakes for Koreans” [17]. If the individual EAA intake of the participants met the RNI, one point was given to each EAA, and the total EAAS was distributed from 0 to 9 points.

### 2.4. Anthropometric and Metabolic Risk Factors

In the health examination information, indicators related to metabolic diseases such as body mass index (BMI), waist circumference (WC), triglyceride (TG), total cholesterol, Low Density Lipoprotein (LDL) cholesterol, High Density Lipoprotein (HDL) cholesterol, systolic blood pressure, diastolic blood pressure, and fasting blood glucose were collected [18]. The BMI was calculated by dividing the body weight (kg) by the square of the height (m^2^). WC was measured at the midpoint between the bottom of the last palpable rib and the top of the iliac crest using a stretch-resistant tape.

All blood samples were measured after 8 h of fasting and were analyzed within 24 h after isolation. Fasting plasma glucose was measured by the hexokinase ultraviolet method using a Tosoh G8 (Tosoh, Tokyo, Japan). Total cholesterol, TG, LDL cholesterol, and HDL cholesterol levels were measured by an enzymatic method using a Labospect008AS (Hitachi, Tokyo, Japan) [18]. For systolic and diastolic blood pressure, three measurements were taken on the right arm using a mercury sphygmomanometer (Baumanmeter^®^ Desk Model 0320; WA Baum, New York, NY, USA), with the average value of the second and third measurements recorded.

MetS was defined based on the National Cholesterol Education Program Adult Treatment Panel III amendment and WC criteria for abdominal obesity presented by the World Health Organization [19]. MetS was confirmed when three or more of the diagnostic criteria for risk factors were met. Each risk factor is expressed as follows: abdominal obesity: WC ≥90 cm in men and ≥80 cm in women [20]; hypertriglyceridemia: TG level ≥150 mg/dL or lipid-lowering medication use; hypo-HDL-cholesterolemia: HDL cholesterol <40 mg/dL in men and <50 mg/dL in women; high blood pressure: systolic blood pressure ≥130 mm Hg, or diastolic blood pressure ≥85 mm Hg, or antihypertensive medication use; and high fasting glucose: fasting glucose ≥100 mg/dL or use of medication for diabetes (insulin or oral agents).

### 2.5. Statistical Analysis

We analyzed data from the 2008–2019 KNHANES, considering multistage, stratified, and clustered sampling methods by applying the KNHANES’ complex sampling design [12]. The characteristics of participants according to the total EAAS were presented as frequencies and percentages for categorical variables using the chi-squared test and as means and standard errors for continuous variables using a linear regression model. The association between the total EAAS and MetS was evaluated using odds ratios (ORs) and 95% confidence intervals (CIs) obtained by multivariate logistic regression analysis. The *p* for trend was calculated using the median values for the quartiles of the total EAAS. Potential effect modifiers were examined using the multiplicative term of the statistical model, and no significant effect modifiers were observed. Potential confounding factors were identified based on preliminary analysis and a comprehensive literature review that investigated the association between amino acid intake and MetS [21,22]. To adjust for confounding factors clearly and systematically, a step-by-step model was used as follows: Model 1, unadjusted; Model 2, adjusted for age and sex; and Model 3, adjusted for age, sex, education level, household income, alcohol consumption, smoking status, BMI, physical activity, and total energy intake. To assess the non-linearity between the total EAAS and MetS, restricted cubic spline regression analysis was conducted. Three knots were used for the analysis, and adjustments were made for the same covariates used in Model 3. All statistical analyses were performed using the Statistical Analysis System (SAS) ver. 9.4 (SAS Institute, Cary, NC, USA), and the statistical significance level for all assessments was set at α = 0.05.

## 3. Results

The general characteristics of the participants according to the total EAAS quartiles are presented in Table 1. The median total EAAS for quartiles 1, 2, 3, and 4 were 6.3, 8.0, 8.6, and 9.0, respectively. Higher levels of total EAAS were associated with men (*p* < 0.001) and younger age groups (*p* < 0.001). In addition, higher total EAAS was associated with higher education levels (*p* < 0.001), household income (*p* < 0.001), and alcohol consumption (*p* < 0.001). The percentage of smokers in the highest EAAS group was approximately 4% greater than that in the group with the lowest total EAAS (*p* < 0.001). The proportion of participants who were physically active was 32.00%, 32.75%, 33.76%, and 34.78% in the 1st, 2nd, 3rd, and 4th EAAS quartile, respectively (*p* < 0.001).

Table 2 presents an adjusted comparison of the metabolic biomarker levels according to the total EAAS. TG levels were significantly lower in those with an increased total EAAS (*p* for trend = 0.002). Systolic and diastolic blood pressure showed similar patterns. As the total EAAS increased, the systolic blood pressure was 118.26 ± 0.24 mm Hg, 117.17 ± 0.23 mm Hg, 116.64 ± 0.22 mm Hg, and 116.04 ± 0.25 mm Hg, and the diastolic blood pressure was 76.80 ± 0.17 mm Hg, 76.33 ± 0.16 mm Hg, 76.14 ± 0.15 mm Hg, and 75.64 ± 0.18 mm Hg (all, *p* for trend < 0.001).

Table 3 shows the association between the total EAAS and MetS. In the fully adjusted Model 3, the prevalence of MetS was 14% lower among participants in the highest quartile of the EAAS than among those in the lowest quartile (OR: 0.86, 95% CI: 0.74–0.996), and an inverse linear association was observed (*p* for trend = 0.03). Compared with the lowest EAAS quartile in Model 3, participants in the highest EAAS quartile were less likely to have high blood pressure (OR: 0.86, 95% CI: 0.75–0.98, *p* for trend = 0.03) and hypertriglyceridemia (OR: 0.86, 95% CI: 0.76–0.98, *p* for trend = 0.047). There was no significant association between the total EAAS and hyperglycemia (*p* for trend = 0.7), although the prevalence of hyperglycemia was significantly lower in the 2nd quartile of EAAS (OR: 1.19, 95% CI: 1.06–1.33).

**Figure 1:**
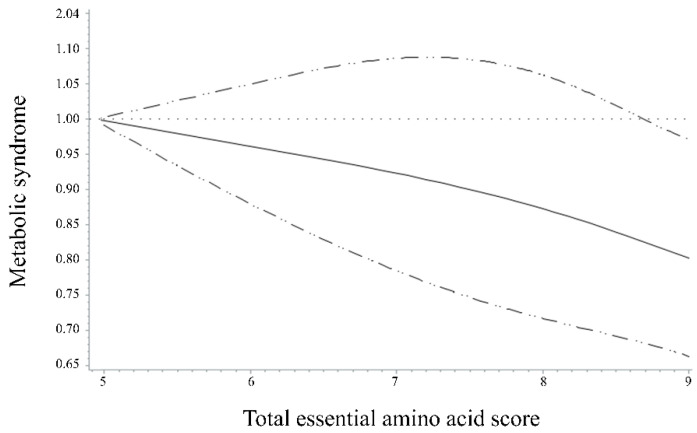
Odds ratios (95% confidence intervals) for the non-linear relationship between the total essential amino acid score and metabolic syndrome, evaluated with restricted cubic splines. Solid line indicates the odds ratios and dotted lines indicate the 95% confidence intervals. The model was adjusted for age, sex, education level, household income, alcohol consumption, smoking status, BMI, physical activity, and total energy intake. *p* for nonlinearity = 0.6.

Figure 1 shows the spline curves representing the linear relationship between the total EAAS and MetS. The model was adjusted for age, sex, education level, household income, alcohol consumption, smoking status, BMI, physical activity level, and total energy intake. The model shows that the test for non-linearity was not significant (*p* for non-linearity = 0.6), indicating that the association between the total EAAS and MetS was linear.

## 4. Discussion

Our hypothesis was verified that higher total EAAS was significantly associated with a lower prevalence of various forms of metabolic dysregulation, including high blood pressure, hypertriglyceridemia, and MetS. In addition, there was an inverse dose–response relationship between the total EAAS and MetS.

Unlike non-EAAs, EAAs are amino acids that are not synthesized in the body and must be obtained from the diet [23]. EAAs are known to play an important role in synthesizing proteins and are involved in the regeneration of muscle cells [23] and the regulation of blood glucose and lipid metabolism [24]. Several amino acid sensors, including general control nonderepressible 2 (GCN2), activating transcription factor 4 (ATF4), mammalian target of rapamycin (mTOR), and AMP-activated protein kinase (AMPK), enable cells to properly respond to changes in amino acid levels to maintain metabolic homeostasis and play a pivotal role in lipid metabolism, glucose metabolism, and energy homeostasis [25]. First, GCN2 is recognized as a sensor for amino acid deficiency, phosphorylates eukaryotic initiation factor 2-α (eIF2α), inhibits general protein synthesis, and increases the translation of proteins involved in amino acid biosynthesis and transport [26]. mTORC1 is specifically regulated by leucine [27], and intraventricular injection of leucine can activate mTOR in the hypothalamus, which is involved in regulating food intake [28]. In addition, ATF4 plays an important role in the regulation of high levels of proliferation required during fetal liver hematopoiesis [29], organ memory [30], osteoblast differentiation [31], endoplasmic reticulum stress [32,33], glucose metabolism, and energy homeostasis [34]. AMPK is an important cellular energy sensor and signal transducer for maintaining energy homeostasis, a process regulated by extensive metabolic stress [35,36] and which plays a role in regulating insulin sensitivity by directly phosphorylating insulin receptor substrate 1 [37] or by phosphorylating tuberous sclerosis complex 2, an upstream inhibitor of mTOR [38]. Currently, no clear mechanism has been identified for how the body detects amino acid levels and regulates metabolic homeostasis, including lipid metabolism, glucose metabolism, and energy homeostasis, but it could be assumed that amino acid sensors have a role in metabolic diseases [25].

We observed that participants who had a high EAAS, showing that the overall EAA intake met the RNI, were less likely to have prevalent MetS and its components. Our results are in line with those of a previous study in which a higher intake level of dietary EAAs was significantly associated with decreased cardiovascular disease mortality in the National Health and Nutrition Examination Survey [39]. The Tehran Lipid and Glucose Study of 2369 Iranians aged ≥19 years showed no statistically significant association between dietary EAAs and cardiovascular disease incidence, but showed an inverse relationship [40].

We did not observe a significant association between the EAAS and HDL cholesterol in this population. In general, Koreans consume high-carbohydrate and low-fat diets that are closely associated with an elevated TG level and a reduced HDL cholesterol level, which are the most common indicators of MetS in this population [41]. Therefore, we speculate that an increased EAA intake may be associated with a decreased proportion of energy from carbohydrates but not strongly associated with fat intake; thus, a higher EAAS may effectively enhance TG but not HDL cholesterol [42].

Several previous studies have reported the effects of specific individual amino acid supplementation on MetS and its components [43,44]. However, intensive supplementation of specific individual amino acids can lead to absorption competition between amino acids [45], resulting in an amino acid imbalance and toxicity risk [46]. Thus, excessive intake of supplements may be inappropriate. To efficiently synthesize proteins in cells, it is imperative to balance the intake of all EAAs. Furthermore, the main food sources of EAAs in Koreans are grains and grain products, which compose the diet at a much higher proportion than the next most common source, seafood and meat [47]. In relation to this fact, an earlier study demonstrated the importance of consuming high-quality protein sources with better biological value [48]. Therefore, it is important to ensure that the RNI is met through a well-balanced intake of protein and non-protein sources of total EAAs.

This study has several limitations. First, although we attempted to adjust for all potential confounding factors, there may have been residual confounding factors that were not accounted for due to the nature of the study. Second, KNHANES is a cross-sectional study in which the EAA intake information and MetS information were collected at the same time. Thus, we cannot clearly identify a causal relationship between EAA intake and the prevalence of MetS or whether participants with MetS had a high intake of EAAs. Third, since the diet of participants was evaluated using 24 h recall data for a single day, it is possible that the usual amount of food intake was not reflected. However, to minimize this limitation, a trained examiner obtained the dietary information using standardized protocols, and participants who showed extreme levels of energy consumption (less than 500 kcal or more than 5000 kcal) and those who reported that their reported dietary information was more or less than their usual diet were excluded. Fourth, there is a possibility that the level of amino acid intake may have been underestimated, as there may be foods with amino acid content values that are unaccounted for in the food database. However, to compensate for this limitation, we aimed to supplement missing nutrients by establishing protocols such as considering alternative foods and applying moisture coefficients. Despite these limitations, this study calculated the total EAAS using KNHANES, a large-scale national sample survey representing Koreans, and analyzed the association between MetS and its components based on the total EAAS. To the best of our knowledge, this study is the first to assess the association between total EAA intake and MetS in Korean adults. In the future, data from this study are expected to serve as the basis for future studies.

## 5. Conclusions

In conclusion, this study shows that the prevalence of MetS was lower when the overall intake of all individual EAAs reached the RNI in Korean adults aged ≥30 years. This suggests the importance of sufficient intake of all EAAs to meet the RNI. This study is expected to be used as a scientific basis for the importance of EAAs in the management of MetS in Korean adults. Future large-scale prospective cohort and clinical trial studies should be conducted to clarify the causal relationship between EAA intake and MetS.

## Figures and Tables

**Table 1:** General characteristics of the participants according to quartile of total EAAS, KNHANES 2008–2019 (*n* = 25,787).

	Total EAAS				*p* ^1^
	Q1	Q2	Q3	Q4	
	*n* = 6446	*n* = 6447	*n* = 6447	*n* = 6447	
Score, Median (range)	6.3 (0.5–7.4)	8.0 (7.5–8.2)	8.6 (8.3–8.8)	9.0 (8.9–9.0)	
Age (years)	54.03 ± 0.16	51.56 ± 0.16	49.28 ± 0.16	48.05 ± 0.16	<0.001
Sex					<0.001
Men	2313 (35.88)	2504 (38.84)	2750 (42.66)	3073 (47.67)	
Women	4133 (64.12)	3943 (61.16)	3697 (57.34)	3374 (52.33)	
Education level					<0.001
Lower than high school education	2708 (44.50)	2083 (34.09)	1574 (25.59)	1290 (20.85)	
High school educated or higher	3377 (55.50)	4028 (65.91)	4578 (74.41)	4898 (79.15)	
Household income					<0.001
Lower or Mid-low	3319 (52.19)	2657 (41.52)	2321 (36.28)	2150 (33.63)	
Mid-high or Higher	3041 (47.81)	3742 (58.48)	4077 (63.72)	4243 (66.37)	
Alcohol consumption					<0.001
Drinkers	4254 (68.23)	4470 (71.22)	4760 (76.00)	4922 (78.15)	
Non-drinkers	1981 (31.77)	1806 (28.78)	1503 (24.00)	1376 (21.85)	
Smoking status					<0.001
Smokers	1160 (18.60)	1113 (17.72)	1259 (20.08)	1417 (22.47)	
Non-smokers	5078 (81.40)	5168 (82.28)	5012 (79.92)	4888 (77.53)	
Body mass index (kg/m^2^)	23.36 ± 0.04	23.40 ± 0.04	23.35 ± 0.04	23.50 ± 0.04	0.08
Physical activity ^2^					<0.001
Low	2154 (35.35)	2036 (33.27)	1998 (32.47)	1926 (31.12)	
Mid	1989 (32.64)	2080 (33.99)	2078 (33.77)	2110 (34.10)	
High	1950 (32.00)	2004 (32.75)	2077 (33.76)	2152 (34.78)	

EAAS, Essential Amino Acid Score; KNHANES, Korea National Health and Nutrition Examination Survey; Q, Quartile. Values are mean ± standard error or *n* (%). ^1^ *p* values were derived from χ^2^ test for categorical variables, and *p* for trends across the quartile of EAAS were calculated using linear regression models for continuous variables. ^2^ Physical activity was categorized into 3 groups, according to tertiles of metabolic equivalents (METs)-hours/week.

**Table 2:** Adjusted average levels of metabolic biomarkers according to the total EAAS, KNHANES 2008–2019 (*n* = 25,787).

	Total EAAS				*p* for Trend
	Q1	Q2	Q3	Q4	
	*n* = 6446	*n* = 6447	*n* = 6447	*n* = 6447	
Score, median (range)	6.3 (0.5–7.4)	8.0 (7.5–8.2)	8.6 (8.3–8.8)	9.0 (8.9–9.0)	
Metabolic biomarkers					
Body mass index (kg/m^2^)	23.63 ± 0.09	23.58 ± 0.08	23.46 ± 0.08	23.44 ± 0.09	0.07
Waist circumference (cm)	81.46 ± 0.16	81.46 ± 0.13	81.11 ± 0.13	81.03 ± 0.16	0.09
Triglyceride (mg/dL)	144.55 ± 2.35	139.92 ± 1.72	134.03 ± 1.60	133.99 ± 2.33	0.002
Total cholesterol (mg/dL)	195.77 ± 0.62	195.97 ± 0.55	195.32 ± 0.53	196.25 ± 0.63	0.6
HDL-cholesterol (mg/dL)	49.99 ± 0.20	50.11 ± 0.18	50.53 ± 0.18	50.57 ± 0.21	0.1
LDL-cholesterol (mg/dL)	119.42 ± 0.98	118.79 ± 0.84	118.74 ± 0.86	119.23 ± 0.98	0.9
Systolic blood pressure (mm Hg)	118.26 ± 0.24	117.17 ± 0.23	116.64 ± 0.22	116.04 ± 0.25	<0.001
Diastolic blood pressure (mm Hg)	76.80 ± 0.17	76.33 ± 0.16	76.14 ± 0.15	75.64 ± 0.18	<0.001
Fasting blood glucose (mg/dL)	97.04 ± 0.32	96.98 ± 0.25	96.45 ± 0.23	96.17 ± 0.30	0.2

EAAS, Essential Amino Acid Score; KNHANES, Korea National Health and Nutrition Examination Survey; Q, Quartile; HDL, High Density Lipoprotein; LDL, Low Density Lipoprotein. Values are presented means ± standard error adjusted for age, sex, and total energy intake.

**Table 3:** The odds ratios and 95% confidence intervals for metabolic syndrome and its components according to the total EAAS, KNHANES 2008–2019 (*n* = 25,787).

	Total EAAS				*p* for Trend
	Q1	Q2	Q3	Q4	
	*n* = 6446	*n* = 6447	*n* = 6447	*n* = 6447	
Metabolic syndrome					
Case	1648	1514	1335	1384	
Model 1	1	0.92 (0.83–1.02)	0.79 (0.72–0.87)	0.82 (0.75–0.90)	<0.001
Model 2	1	0.95 (0.86–1.05)	0.85 (0.77–0.94)	0.90 (0.81–0.99)	0.003
Model 3	1	0.97 (0.86–1.10)	0.87 (0.76–0.998)	0.86 (0.74–0.996)	0.03
Abdominal obesity					
Case	2311	2178	1966	2015	
Model 1	1	0.95 (0.86–1.03)	0.80 (0.73–0.87)	0.84 (0.77–0.92)	<0.001
Model 2	1	0.996 (0.91–1.09)	0.89 (0.81–0.97)	0.99 (0.90–1.08)	0.2
Model 3	1	1.05 (0.90–1.23)	0.93 (0.79–1.09)	0.96 (0.79–1.16)	0.5
Hyperglycemia					
Case	1696	1756	1639	1661	
Model 1	1	1.12 (1.02–1.23)	1.02 (0.93–1.12)	0.99 (0.90–1.09)	0.9
Model 2	1	1.15 (1.04–1.26)	1.07 (0.97–1.18)	1.04 (0.94–1.15)	0.3
Model 3	1	1.19 (1.06–1.33)	1.08 (0.95–1.22)	0.95 (0.83–1.09)	0.7
High blood pressure					
Case	1861	1689	1572	1568	
Model 1	1	0.90 (0.82–0.99)	0.87 (0.79–0.95)	0.86 (0.78–0.94)	<0.001
Model 2	1	0.93 (0.84–1.02)	0.92 (0.83–1.01)	0.91 (0.83–1.01)	0.048
Model 3	1	0.91 (0.82–1.02)	0.91 (0.81–1.02)	0.86 (0.75–0.98)	0.03
Hypo-HDL-cholesterolemia					
Case	2607	2414	2257	2160	
Model 1	1	0.91 (0.84–0.996)	0.78 (0.72–0.85)	0.75 (0.69–0.82)	<0.001
Model 2	1	0.95 (0.87–1.04)	0.85 (0.78–0.93)	0.86 (0.79–0.94)	<0.001
Model 3	1	1.00 (0.91–1.10)	0.91 (0.82–1.01)	0.96 (0.86–1.08)	0.2
Hypertriglyceridemia					
Case	1961	1894	1871	1924	
Model 1	1	0.98 (0.90–1.08)	0.95 (0.87–1.04)	0.98 (0.90–1.08)	0.5
Model 2	1	0.96 (0.88–1.06)	0.92 (0.84–1.01)	0.92 (0.84–1.01)	0.04
Model 3	1	0.96 (0.87–1.07)	0.93 (0.83–1.04)	0.86 (0.76–0.98)	0.047

EAAS, Essential Amino Acid Score; KNHANES, Korea National Health and Nutrition Examination Survey; Q, Quartile; HDL, High-Density Lipoprotein. Model 1: unadjusted. Model 2: adjusted for age, and sex. Model 3: adjusted for age, sex, education level, household income, alcohol consumption, smoking status, BMI, physical activity, and total energy intake.
